# Synthesis and Characterization of Gold Chiral Nanoparticles Functionalized by a Chiral Drug

**DOI:** 10.3390/nano13091526

**Published:** 2023-04-30

**Authors:** Simona Bettini, Michela Ottolini, Donato Valli, Rosanna Pagano, Chiara Ingrosso, Maarten Roeffaers, Johan Hofkens, Ludovico Valli, Gabriele Giancane

**Affiliations:** 1 Department of Biological and Environmental Sciences and Technologies, University of Salento, Via per Monteroni, 73100 Lecce, Italy; 2 Department of Engineering for Innovation, University of Salento, Via per Monteroni, 73100 Lecce, Italy; 3 Department of Chemistry, KU Leuven, Celestijnenlaan 200F, 3001 Leuven, Belgium; 4 CNR-IPCF SS Bari, c/o Dipartimento di Chimica dell’Università degli Studi di Bari, Via Orabona 4, 70126 Bari, Italy; 5 cMACS, KU Leuven, Celestijnenlaan 200F, 3001 Leveun, Belgium; 6 Department of Cultural Heritage, University of Salento, Via D. Birago 84, 73100 Lecce, Italy

**Keywords:** gold nanoparticles, chiral nanostructures, D-penicillamine, spectroscopic characterization, circular dichroism

## Abstract

Inorganic chiral nanoparticles are attracting more and more attention due to their peculiar optical properties and potential biological applications, such as bioimaging, therapeutics, and diagnostics. Among inorganic chiral nanoparticles, gold chiral nanostructures were demonstrated to be very interesting in this context, with good physical chemical stability and also the possibility to decorate the surface, improving biomedical application as the interaction with the bio-systems. Gold (Au) nanostructures were synthesized according to a seed-mediated procedure which envisages the use of cetyltrimethylammonium bromide (CTAB) as the capping agent and L- and D-cysteine to promote chirality. Au nanostructures have been demonstrated to have opposite circular dichroism signals depending on the amino acid enantiomer used during the synthesis. Then, a procedure to decorate the Au surface with penicillamine, a drug used for the treatment of Wilson’s disease, was developed. The composite material of gold nanoparticles/penicillamine was characterized using electron microscopy, and the penicillamine functionalization was monitored by means of UV-Visible, Raman, and infrared spectroscopy, highlighting the formation of the Au–S bond. Furthermore, electron circular dichroism was used to monitor the chirality of the synthesized nanostructures and it was demonstrated that both penicillamine enantiomers can be successfully bonded with both the enantiomers of the gold nanostructures without affecting gold nanoparticles’ chirality. The effective modification of nanostructures’ surfaces via penicillamine introduction allowed us to address the important issue of controlling chirality and surface properties in the chiral nano-system.

## 1. Introduction

Chirality is a physical–chemical phenomenon characterizing all the natural and biological systems from the intramolecular to the supramolecular scale [1]. Basically, two enantiomers of a chiroptical compound are characterized by the capability to rotate, in the opposite direction, the versus of rotation of circularly (clockwise or anticlockwise) polarized incident light [2]. This feature confers them peculiar optical activities, such as circular dichroism, circularly polarized luminescence, and non-linear optics [3]. Biological and physiological processes are basically stereoselective, being based on chiral molecules and/or chiral building blocks (amino acids, sugars) [4]. Taking inspiration from this aspect, in recent years, researchers have focused their attention on the synthesis of chiral inorganic nanostructures with the aim to combine the outstanding (bio)technological applications of nanoparticles (NPs) with the intriguing and multifaceted potentials of chiral systems. Thus, chiral inorganic NPs should provide similarities with the molecular building blocks normally present in the organisms in a completely bio-inspired frame being, so that they are suitable for different biological and biomedical applications, such as imaging, diagnostics, biosensing, and therapeutics [5].

Not less important, NPs can be easily functionalized by surface ligands and the introduction on NPs’ surface of specific ligands of biological interest plays a major role in the subsequent bio-applications [6]. Surface ligands, in fact, affecting surface properties, can be used to improve interactions with the biosystems (such as cells and bacteria) providing higher biocompatibility, cellular uptake and stability [7], and improving the final application. For instance, it was demonstrated that the chirality of the structures strongly influences their biological application [8,9,10], such as cell differentiation [11] and cellular uptake [12]. In fact, it is reported that achiral structures decorated with chiral molecules are internalized in the cells with a marked enantio-specificity [13,14]. More recently, as well as the nanoparticles functionalized with organic chiral molecules, it was demonstrated that chiral-selectivity rules the cellular uptake of chiral inorganic structures, too [15].

More generally, among inorganic nanoparticles, plasmon metal nanoparticles and their synthesis and functionalization are a hot topic in physical sciences, chemistry, and in material science [16,17]. The interest in these nanostructures rises from the manifold and multi-faceted applications where they can be applied: devices for energy harvesting [18], active platforms for environmental issues [19,20], opto-electronic devices [21,22,23], sensors [24,25,26], and biological applications [27]. Among the plasmonic materials, gold nanoparticles (AuNPs) show several advantages if compared with other plasmonic nanostructures. For example, they are stable, they can be obtained by means of biological approaches [28], they are not toxic to human beings and animals [29], and they can be functionalized by different compounds and molecules for pharmacological tests [30], as well as sensor and opto-electronic applications [31,32].

In addition to the straightforwardness of functionalizing AuNPs with interesting biomolecules, the naked gold nanoparticles can be efficiently uptaken by living cells [33]. The AuNPs’ cellular internalization is influenced by the nanoparticle dimension, by the shape, by the surface charge [33,34,35], and by the molecules and biomolecules used to decorate the AuNPs’ surface [36]. For synthetizing chiral AuNPs, it is possible to list three main approaches: the synthesis of metal nanoparticles with intrinsic chiral morphology, the complexation of chiral molecules and nanoparticles, and the chiral arrangement of nanoparticles [37,38,39].

**Scheme 1:**
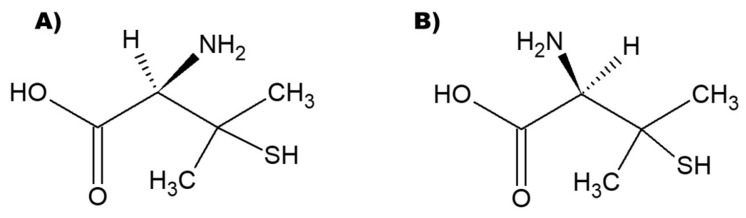
Chemical structures of the two enantiomers of penicillamine (**A**) D enantiomer and (**B**) L enantiomer.

In the present work, Au nanoparticles with intrinsic chiral morphology mainly based on rhombic dodecahedrons were synthesized and were further functionalized using a chiral molecule that was demonstrated as very interesting from a pharmacological point of view, that is, penicillamine (Scheme 1).

Penicillamine is used for the treatment of Wilson’s disease, a clinical condition that derives from an over-accumulation of copper in the human body, in particular in the liver and brain [40]. It causes several and severe health problems such as rheumatoid arthritis, hepatic impairment, and neurological disorders. Penicillamine is reported as a heavy metal antagonist drug and D-penicillamine is largely used to chelate copper ions forming D-penicillamine: Cu = 2:1 complex [41]. As typically observed when chiral drugs are used, the L enantiomer of penicillamine’s effects on human health are considered dangerous; L-penicillamine is not clinically used due to its strong inhibition of pyridoxine-dependent enzymes, leading to neurotoxicity in animal experiments [42], and D-penicillamine is exclusively used for the treatment of Wilson’s disease and other clinical treatments.

Therefore, in this contribution, we prepared chiral gold nanostructures exploiting a seed-mediated procedure to produce gold nanostructure seeds further treated in a growth solution containing L- and D-cysteine to drive the formation of chiral helicoidal nanostructures [39,43,44,45]. Then, a capping agent exchange easy procedure was developed to introduce D-penicillamine, preserving the chirality of the starting nanoparticles.

## 2. Materials and Methods

### 2.1. Materials

Hexadecyltrimethylammonium bromide (CTAB, 98%), L-ascorbic acid (AA, 99%), sodium borohydride (NaBH_4_, 98%), gold(III) chloride trihydrate (HAuCl_4_·3H_2_O, 99.9%), L-cysteine hydrochloride monohydrate (98%), D-cysteine hydrochloride monohydrate (95%), and D-penicillamine (98–101%) were purchased from Merck and used as received without any further purification. All solutions were prepared in ultrapure water (18 MΩ cm).

### 2.2. Chiral AuNP Synthesis Procedure

Chiral AuNPs were synthesized according to a seed-mediated reported procedure [44,45] with slight modifications. In a typical experiment, a first spherical Au seed was prepared by mixing saturated (or at least above the critical micellar concentration) CTAB solution and 10 mM HAuCl_4_ in 7.75 mL final volume under gentle stirring. Then, 0.8 mL of 10 mM NaBH_4_ was added dropwise and the reaction was left for 3 h at room temperature. In the second step, growth envisaged the dispersion of 0.2 mL of 10 mM HAuCl_4_ in 1.6 mL of saturated CTAB aqueous solution and was then diluted in 8 mL of ultrapure water. A total of 0.95 mL of L-AA (50 mM) and 0.01 mL of the first seed solution were finally added to the mixture, which was left for 45 min and then washed twice via centrifugation at 5000 rpm for 5 min. The produced seeds (Au@CTAB) were redispersed in CTAB (1 mM). The third step of the proposed synthesis procedure allowed the fabrication of helicoidal chiral AuNP, L-AuNH@CTAB, and D-AuNH@CTAB: 0.1 mL of HAuCl_4_ (10 mM) was dispersed in 1.6 mL of saturated CTAB solution and then diluted in 7.9 mL of ultrapure water. Then, 0.95 mL of L-AA (0.1 M) and 0.01 mL of 0.1 mM Cys (L or D) were added. Finally, 0.1 mL of the second Au seeds was added. The reaction was carried out at 37 °C for 30 min, then chiral AuNPs were centrifuged twice (5000 rpm, 5 min) and concentrated in 2 mL of 1 mM CTAB solution to be stored at room temperature for further experiments.

### 2.3. D- and L-Penicillamine Functionalization of Chiral Gold Nanostructures

1 mL of L-AuNH@CTAB and D-AuNH@CTAB, in four different experiments, was mixed with 1 mL of 10 mM D- and L- penicillamine aqueous solution in order to have a large excess of penicillamine in the mix to induce a capping agent exchange, substituting CTAB with penicillamine to obtain L- and D-AuNH capped with penicilammine. The reaction was carried out under gentle agitation (100 rpm) for 12 h at room temperature. The penicillamine excess was removed via centrifugation (5000 rpm, 5 min), and then L- and D-AuNH capped with penicillamine were washed twice in ultrapure water and finally resuspended in 1 mL as the final volume for the characterization.

### 2.4. Characterization Methods

The samples were investigated by means of UV-Vis spectroscopy (Cary5000, Agilent); Electronic Circular Dichroism (ECD) spectra were recorded using a JASCO (J-1500 CD Spectrometer) with a scanning speed of 20 nm min^−1^; the electrostatic charge of the nanoparticles was monitored by means of a Malvern Panalytical Nano ZS Zetasizer instrument (Malvern Panalytical Limited, Malvern, UK).

Scanning electron microscopy (SEM) images were recorded on a FEI-Q FEG250 instrument.

Transmission electron microscopy (TEM) was used for the morphological characterization of the synthesized nanostructures (Jeol Jem-1011 microscope operating at 100 kV and equipped with a high-contrast objective lens, a W filament as an electron source, with an ultimate point resolution of 0.34 nm). For TEM samples’ preparation, 300-mesh amorphous carbon-coated Cu grids were dipped in water solutions of the AuNPs and the measurements were carried out after water evaporation.

Raman spectroscopy was used to monitor the capping exchange from Hexadecyltrimethylammonium bromide (CTAB) to penicillamine: a 785 nm laser (power 0.125 mW cm^−2^) was used as a luminous source. For this characterization, the samples dispersed in water were drop-casted on silicon substrates and, after the solvent evaporation, Raman spectra with 50 scans and 10 s of integration were recorded using a 10× objective. Raman spectroscopy was performed using a microRaman Xplora Horiba. Fourier Transform Infrared characterization (FTIR) with ATR tool [46] was used for monitoring the presence of CTAB and penicillamine: the AuNPs were deposited directly on ATR crystal via drop-casting and 64 scans in the range 3400–800 cm^−1^ were acquired for each sample using a Perkin-Elmer Spectrum One spectrophotometer.

## 3. Results and Discussion

### 3.1. Spectroscopic and Morphologic Characterization of L- and D-AuNH@CTAB

With the final aim of producing gold nanostructures that were intrinsically chiral but also further decorated with a chiral ligand of biological interest, i.e., penicillamine [40], chiral AuNPs were fabricated by exploiting a slightly modified method reported in the literature [39,44]. The procedure is a two-step seed-mediated protocol which guaranteed the formation of nanostructures with high-Miller-index surfaces characterized by innate chirality due to the atomic arrangement and by enantioselective interactions with chiral ligands [47]. Therefore, during the last step of the synthesis procedure, the adding of L-Cys and D-Cys and the formation of Au–S bonds further contribute to the chirality of the synthesized nanoparticles.

**Figure 1:**
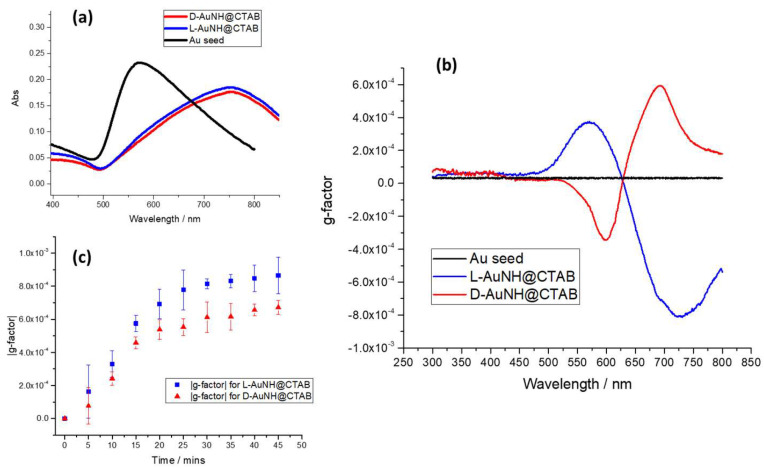
(**a**) UV-Vis spectra and (**b**) ECD spectra of Au@CTAB seed and chiral AuNPs. Black line is the absorption spectrum of the Au seeds, blue line is the profile of the plasmonic peak of L−AuNH@CTAB, and red line is related to D−AuNH@CTAB nanoparticles. (**c**) g-factor intensity of L−AuNH@CTAB and D−AuNH@CTAB was monitored during the experiment at 37 °C in the last step of the synthesis procedure.

The structures obtained after the second step growth, Au@CTAB, were characterized by means of UV-Vis spectroscopy and circular dichroism (black lines in Figure 1a,b). The plasmonic peak was centered at about 570 nm and, as was easily predictable, no dichroic signal was observed.

Particularly interesting is the new profile and position of the plasmonic peak after the synthetic procedure in the presence of the two enantiomers of cysteine. The signals, both for the L-AuNH@CTAB and for D-AuNH@CTAB, are strongly shifted up to 756 nm and the spectral profile appears very similar for both the obtained nanostructures (blue and red line in Figure 1a). On the contrary, ECD spectra clearly suggest that a chiral form is induced by the presence of the two enantiomers of cysteine, and a positive/negative signal is observed for the D-AuNH@CTAB (red line Figure 1b) and negative/positive for L-AuNH@CTAB (blue line) [45]. The not perfectly symmetric profile of the ECD spectra of the two chiral forms is probably a consequence of the L-cysteine (purity ≥ 99%) and D-cysteine (purity 98%) used to promote the chirality in the gold nanostructures. The optical activities of the two species were monitored during the experiment and an optimum value was observed after 30 min of the thermal treatment at 37 °C in both cases (Figure 1c).

**Figure 2:**
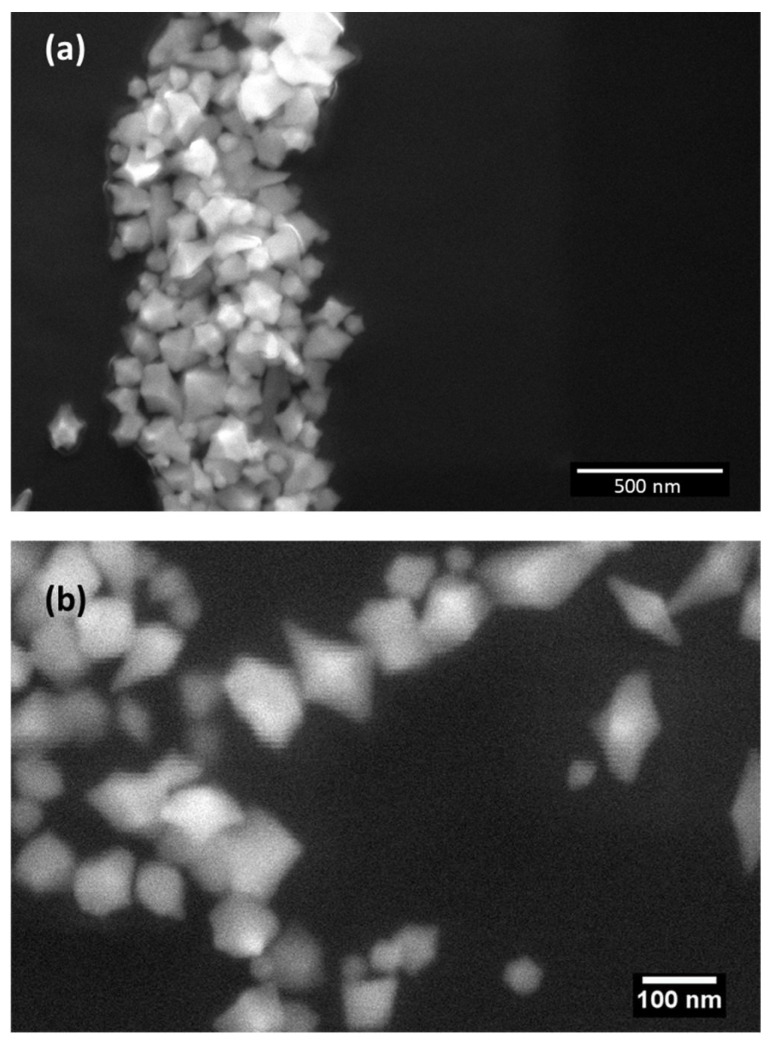
SEM images of D-AuNH@CTAB (image (**a**)) and L-AuNH@CTAB (image (**b**)).

Morphological investigations were performed using SEM and TEM. In particular, SEM images of both D- and L-AuNH@CTAB (Figure 2a,b) showed multifaced tridimensional structures comprising rhombic dodecahedron shapes in the range 150–300 nm (Figure S1) [45]. TEM images (Figure S2) confirm the reported morphologies already highlighted by SEM, and they appear in good agreement with the data reported by Lee and co-workers [39,44], which ascribed such structures to nanoparticles with chiral features induced by the helicoidal geometric patterns.

**Figure 3:**
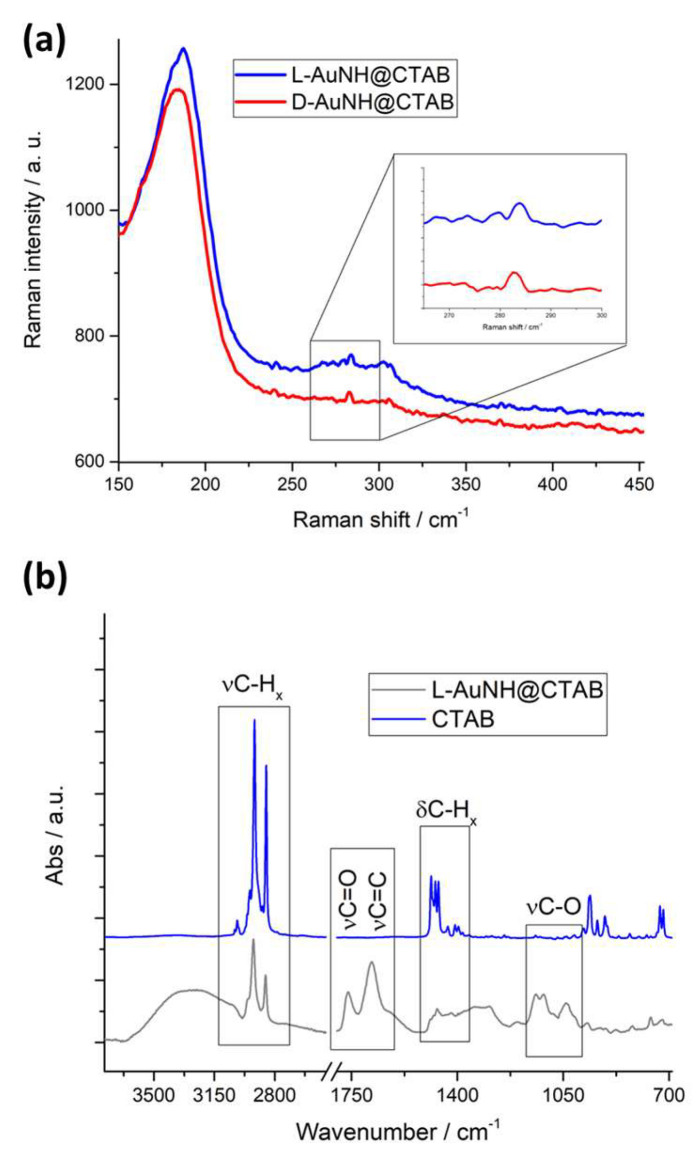
(**a**) Raman spectrum of L−AuNH@CTAB (blue line) and D-AuNH@CTAB (red line) in the region of interest 150–450 cm^−1^. (**b**) FTIR spectra of L−AuNH@CTAB (black spectrum) and CTAB (blue line).

ζ-potential measurements performed on D-AuNH@CTAB and L-AuNH@CTAB revealed a surface positive charge of 53 mV for both nanostructures due to the presence of cetrimonium bromide as the capping agent [48]. The typical Raman spectrum of CTAB-capped AuNPs was obtained for both the gold chiral nanostructures (Figure 3a); the signal at 190 cm^−1^ is indeed ascribable to the formation of a Br–Au bond [49]. Furthermore, it is worth observing that a weak band at about 270 cm^−1^ is observed both in the case of L- and D-AuNH@CTAB nanostructures. Such a small signal, almost negligible if compared with the contributions of the formation of Au-CTAB adduct, is ascribable to the Au–S formation [50].

FTIR spectrum of L-AuNH@CTAB is reported in Figure 3b (black spectrum) and compared with FTIR spectrum of CTAB (blue line). L-AuNH@CTAB and D-AuNH@CTAB are completely superimposable. The signals at 2916 cm^−1^ and 2846 cm^−1^ are ascribable to methyl and methylene CH stretching modes arising from CTAB, and the corresponding bending modes comprise a 1500–1300 cm^−1^ frequency range (zoom in Figure 3b), confirming the presence of CTAB as the main capping agent [51]. Additionally, the contribution of AA on the surface of AuNH is visible. The peaks localized at 1750 cm^−1^ and 1670 cm^−1^ are typical of lactone C=O and C=C stretching modes, and even alcoholic C-O stretching modes are present in the region 1250–1000 cm^−1^ [52].

### 3.2. Spectroscopic and Morphologic Characterization of D-Penicillamine-Capped AuNPs

The chiral gold nanostructures were further manipulated in order to functionalize them by means of the D enantiomer of penicillamine (see Section 2) to obtain L- or D-AuNH@D-Pen. In fact, as reported in the medical literature, L-penicillamine is not used as a pharmacological compound since the side effects due to its consumption cannot be neglected. Nevertheless, the capping exchange was studied even in the presence of L-penicillamine (L- and D-AuNH@L-Pen) for the better understanding of the effect of the different chirality of the capping agent on the intrinsic physical chemical features of the chiral gold nanoparticles. Of course, in both cases, the exchange can be supposed to be driven by Au–S bond formations due to the presence of the thiol group in the chosen compounds [45].

After the capping exchange with D-penicillamine, the ζ-potential reveals that two populations of particles are present: a minority part is represented by AuNPs with a surface charge of 50.8 mV (about 34% of the total population), and the rest shows an electrostatic charge of 37.3 mV (about 66%). It suggests that at least a part of CTAB was substituted with penicillamine on the AuNPs’ surface, reducing the electrostatic charge on the gold nanoparticles. Similar results were obtained using L-penicillamine as the capping agent; in particular, a reduction in the negative charge as a consequence of the CTAB substitution was observed both for L- and D-AuNH@L-Pen nanoparticles (54.8 mV for a population of about 31% and 39.4 mV of about 69% of the total population).

**Figure 4:**
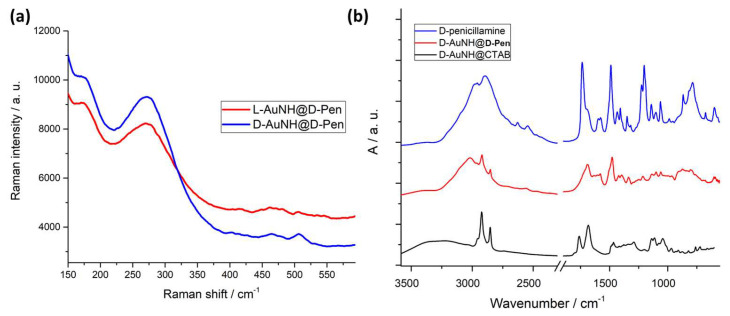
(**a**) Raman spectra of D-AuNH@D-Pen and L-AuNH@D-Pen after the exchange of CTAB with penicillamine; (**b**) comparison of FTIR spectra of penicillamine, D-AuNH@D-Pen, and D-AuNH@CTAB.

As a confirmation of the capping exchange, Raman spectra of both L-AuNH@D-Pen and D-AuNH@D-Pen show an intense signal at 275 cm^−1^ typical of the formation of Au–S interaction [50] (Figure 4a). The band of Au–Br is still evident, even though it is slightly shifted at lower wave numbers. FTIR spectroscopy clearly confirmed the presence of penicillamine on the surface of the nanostructures. In fact, Figure 4 panel b reports the spectra obtained for D-penicillamine (blue line), D-AuNH@D-Pen (red line), and D-AuNH@CTAB (black line), and the presence of both CTAB and penicillamine is undoubtably evident. It is interesting to underline that, according to Raman characterization, the exchange was driven by the Au–S interaction, as expected. The SH stretching modes localized at 2625 cm^−1^ and 2550 cm^−1^ in the penicillamine spectrum undergo a modification in terms of intensity and position in the D-AuNH@D-Pen spectrum.

**Figure 5:**
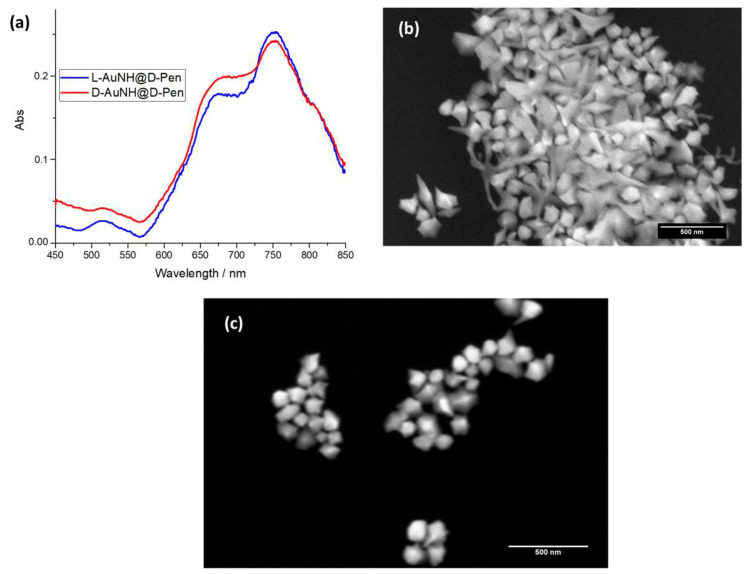
(**a**) UV-Vis spectra of D-AuNH@D-Pen (red line) and L-AuNH@D-Pen (blue line). Some examples of SEM images of D-AuNH@D-Pen (**b**) and L-AuNH@D-Pen (**c**) are reported. The peculiar structures were observed both in D-AuNH@D-Pen and in L-AuNH@D-Pen samples.

The UV-Vis spectroscopy characterization after the capping exchange (reported for L- and D-AuNH@D-Pen in Figure 5) suggests that for both cases, the nanoparticles’ population substantially changes [53]. In fact, the main plasmonic peak appears blue-shifted at about 20 nm, and the other two signals appear at 665 and 520 nm (Figure 5a). This rationale, that a change in shape and/or dimension of D-penicillamine-capped gold nanoparticles takes place, was further confirmed with the SEM and TEM micrographs. In fact, after the capping exchange, it is possible to observe, both in the L- and D-AuNPs’ case, that rhombic-dodecahedron-shaped structures represent the most relevant nanoparticle population, even though elongated structures (of about 400–500 nm length) with defined edges appear for all the samples (Figure 5b,c and Figures S3 and S4).

Upon L-penicillamine exchange, very interestingly, a similar behavior was monitored by means of the morphological characterization (Figure S5) according to ζ-potential measurements. Indeed, at least two morphologies can be identified as ascribable to the rhombic dodecahedrons and to more elongated structures. Such an evidence, again, is confirmed for both the nanoparticles’ enantiomers.

**Figure 6:**
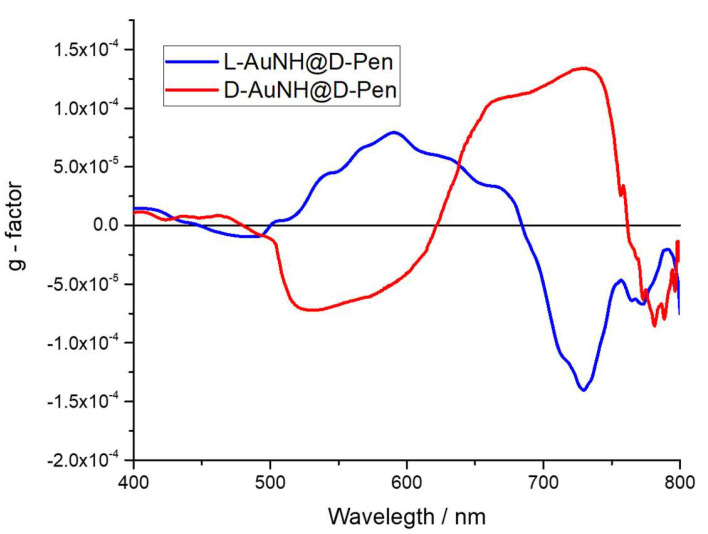
ECD spectra of L-AuNH@D-Pen (blue line) and D-AuNH@D-Pen (red line).

Circular electronic spectra of L-AuNPs and D-AuNPs after the capping exchange with D-penicillamine (Figure 6) show that the two nanostructures preserve the same chirality that was observed before the capping exchange. Furthermore, the ECD profiles of L-AuNH@D-Pen and D-AuNH@D-Pen appear very different: the ECD related to the D-gold nanoparticles is in complete agreement with the UV-Vis spectrum of the same nanostructures: two dichroic signals appear at 730 and 665 nm with the respective Cotton signals. On the contrary, in the case of L-AuNH@D-Pen, a sharp negative signal located at 730 nm suggests that the chirality of the nano-system is still ruled by the particles’ chirality and, very appealing, the interaction of D-penicillamine with the AuNPs is influenced by the gold AuNPs’ chirality.

It can be argued that the functionalization with the chiral ligand on the surface of the chiral nanoparticles does not affect the chirality of the nanostructure itself. According to the literature [44], the chirality of the inorganic plasmonic structures is slightly influenced by the presence of the organic chiral molecules attached to their surface, so the presence of L- and D-penicillamine only influences the g-factor profile without modifying the chirality of the assembly. Additionally, that attachment appears different depending on the chirality of the AuNPs. This means that by fixing the chirality of the AuNPs, the pharmaceutical compound, D-penicillamine, can be easily used to decorate their surface to drive eventual cellular uptake, which will be dependent only on the intrinsic chirality of the nanoparticles, as reported in the literature [15].

The capping exchange process was monitored as g-factor variation during the incubation time, and a slight bleaching of the g-factor was observed after the incubation performed to allow the capping exchange (12 h), in agreement with the literature [44]. In particular, for L-AuNH@D-Pen, a reduction of about 15% (Figure S6c) was observed after 1 h, then a plateau was reached (Figure S6a red triangles). A similar trend was observed for D-AuNH@D-Pen, and a reduction of about 18% (Figure S6c) of the initial g-factor was recorded until reaching the plateau during the monitored reaction time (Figure S6a black triangles). A slight decrease in the g-factor value upon L-penicillamine exchange with L- and D-AuNH was also monitored (Figure S6b).

## 4. Conclusions

In conclusion, we proposed the synthesis of chiral gold nanostructures by means of a well-established procedure based on a seed-mediated synthesis and the use of a chiral amino acid, cysteine, to encode intrinsic chirality. Then, we developed an easy procedure to attach a chiral ligand, L- and D-penicillamine, on the gold surface through Au–S bonds and partially removing the capping agent (CTAB). The final aim was to preserve the obtained gold nanostructures’ intrinsic chirality. In fact, the enantioselective biological and biomedical applications were assessed as stereoselective. In particular, cellular uptake of chiral inorganic nanoparticles is nowadays well-known and, in this frame, our main goal was to achieve the functionalization using a specific enantiomer of a molecule of interest (in this case penicillamine), preserving the AuNPs’ chirality encoded in the first part of the synthesis procedure. Indeed, L- and D-AuNH capped by penicillamine were deeply characterized and we demonstrated carrying out the exchange between CTAB and D- and L-penicillamine while avoiding affecting the overall AuNPs’ chirality.

## Data Availability

Not applicable.

## Supplementary Materials

The following supporting information can be downloaded at: https://www.mdpi.com/article/10.3390/nano13091526/s1, Figure S1: SEM images of (a) D-AuNH@CTAB and (b) L-AuNH@CTAB; Figure S2: TEM images of L-AuNH@CTAB (image a) and D-AuNH@CTAB (image b), Figure S3: SEM images of (a) D-AuNH@D-Pen and (b) L-AuNH@D-Pen, Figure S4: Some examples of TEM images of D-AuNH@D-Pen (images a, b) and L-AuNH@D-Pen (c, d) are reported, Figure S5: SEM images of D-AuNH@L-Pen (a and b) and L-AuNH@L-Pen (c and d), Figure S6: g-factor variation in the capping exchange experiments using (a) D-penicillamine and (b) L-penicillamine; (c) |g-factor|loss (%) vs. reaction time (hours) (|g-factor|loss = $\frac{G_{0}-G_{t}}{G_{0}}\times 100$).
