# Supplementary material for: Synthesis and Characterization of Gold Chiral Nanoparticles Functionalized by a Chiral Drug
Source: Nanomaterials (Basel). 2023 Apr 30;13(9):1526. doi: 10.3390/nano13091526 (PMC10180680; doi:10.3390/nano13091526)
Supplement: Supplementary file 1 [file nanomaterials-13-01526-s001.zip › nanomaterials-2279243-supplementary.pdf]

nanomaterials

Supplementary Materials

Synthesis and Characterization of Gold Chiral Nanoparticles Functionalized by a Chiral Drug

Simona Bettini [1], Michela Ottolini [2], Donato Valli [3], Rosanna Pagano [1], Chiara Ingrosso [4], Maarten Roeffars [5], Johan Hofkens [3], Ludovico Valli [1] and Gabriele Giancane [6,*]

[1] Department of Biological and Environmental Sciences and Technologies, University of Salento, Via per Monteroni, 73100 Lecce, Italy
[2] Department of Engineering for Innovation, University of Salento, Via per Monteroni, 73100 Lecce, Italy
[3] Department of Chemistry, KU Leuven, Celestijnenlaan 200F, 3001 Leuven, Belgium
[4] CNR-IPCF SS Bari, c/o Dipartimento di Chimica dell'Università degli Studi di Bari, Via Orabona 4, 70126 Bari, Italy
[5] cMACS, KU Leuven, Celestijnenlaan 200F, 3001 Leveun, Belgium
[6] Department of Cultural Heritage, University of Salento, Via D. Birago 84, 73100 Lecce, Italy
* Correspondence: gabriele.giancane@unisalento.it; Tel.: +39-0832299442

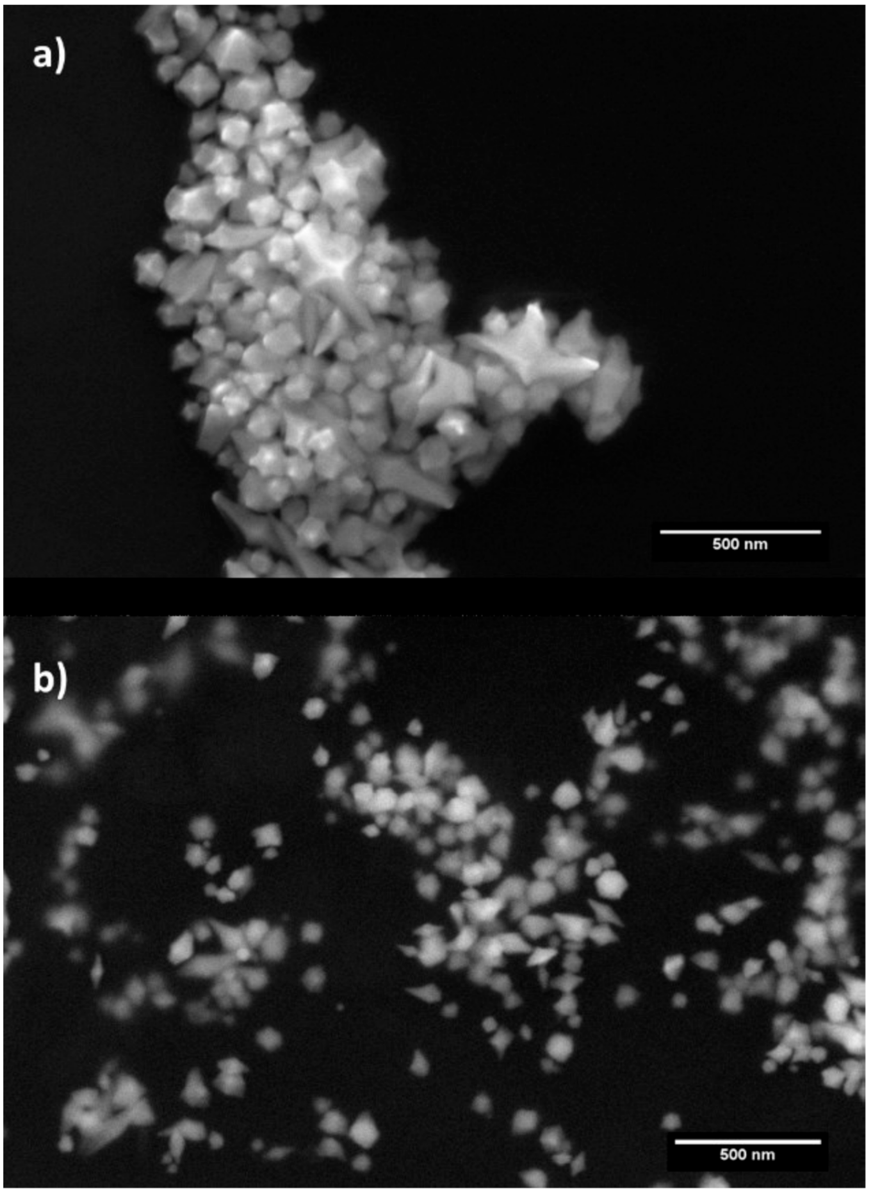

Figure S1. SEM images of **a)** D-AuNH@CTAB and **b)** L-AuNH@CTAB.

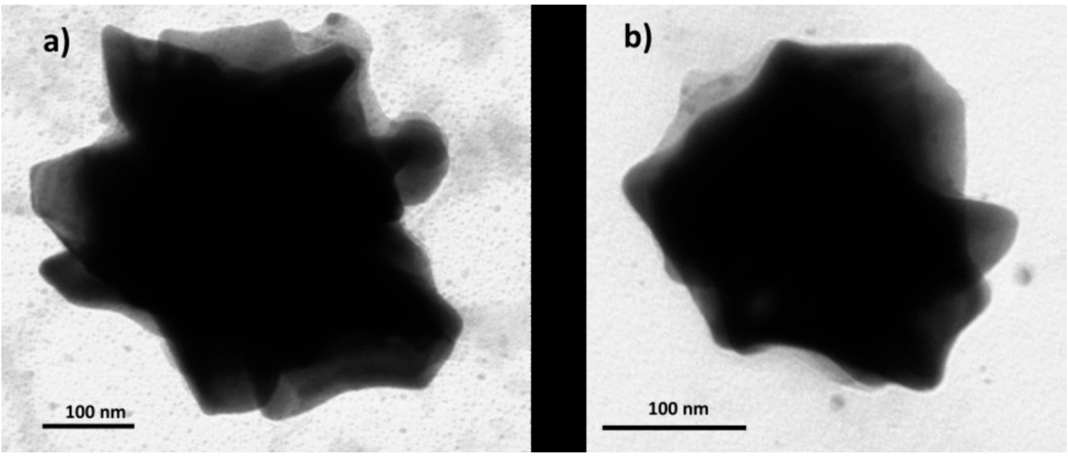

Figure S2. TEM images of L-AuNH@CTAB (**a**) and D-AuNH@CTAB (**b**).

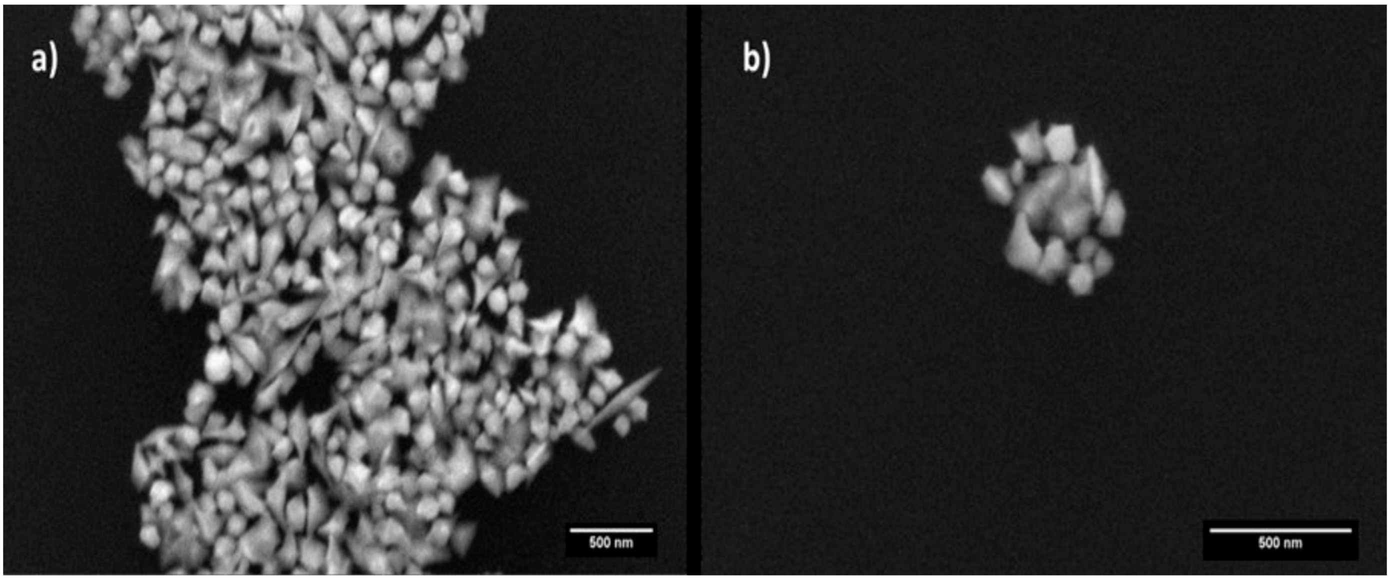

Figure S3. SEM images of (**a**) D-AuNH@D-Pen and (**b**) L-AuNH@D-Pen.

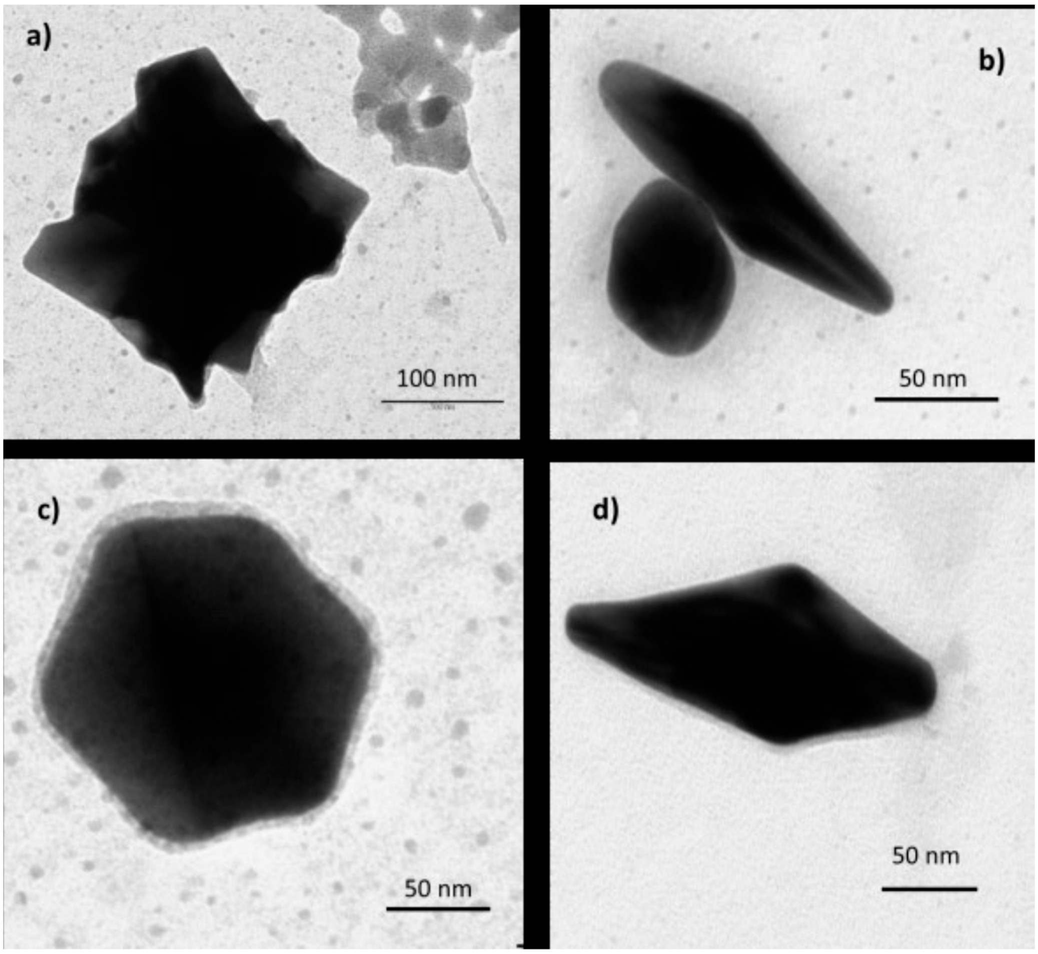

Figure S4. Some examples of TEM images of D-AuNH@D-Pen (**a**,**b**) and L-AuNH@D-Pen (**c**,**d**) are reported.

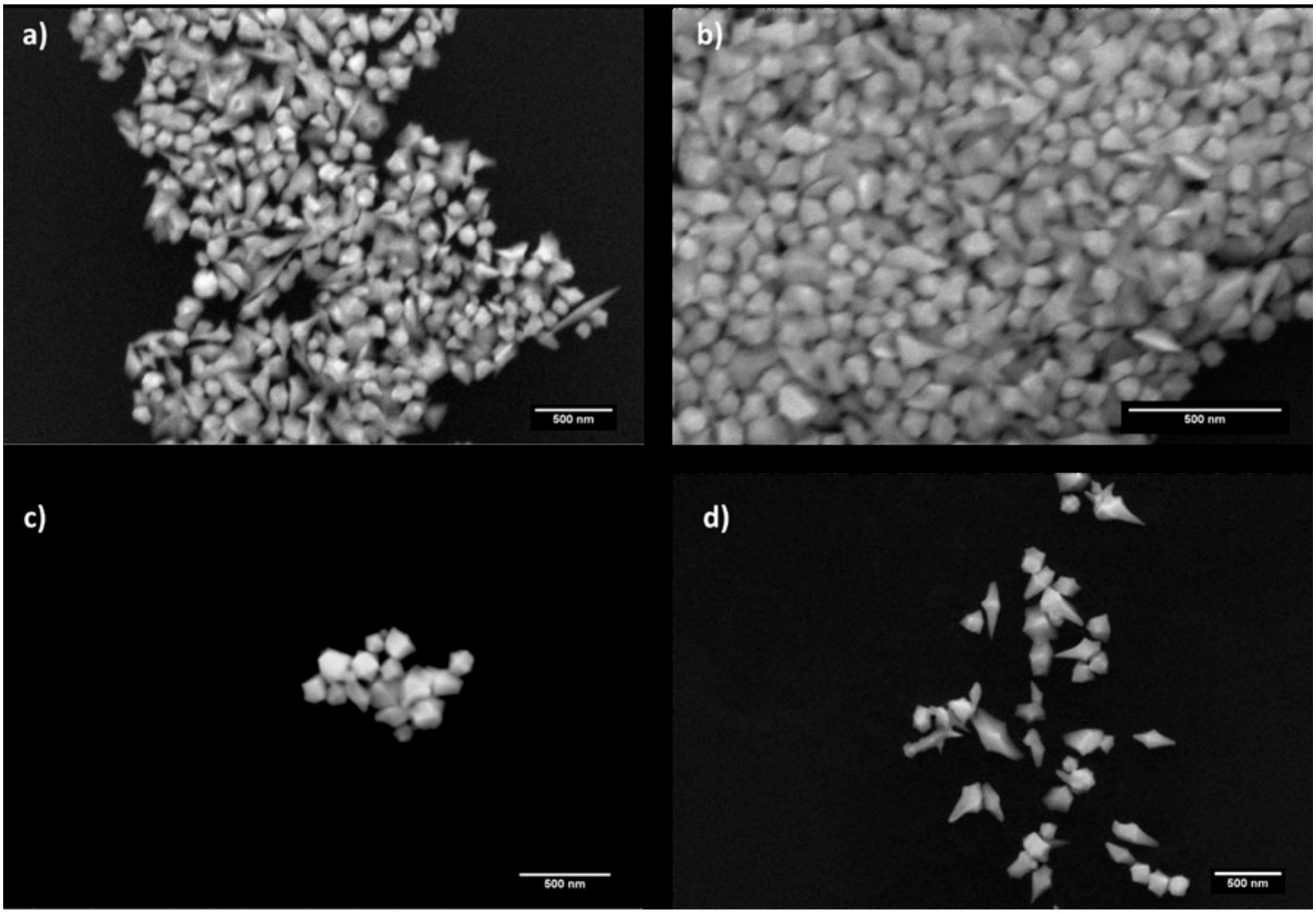

Figure S5. SEM images of D-AuNH@L-Pen (**a**,**b**) and L-AuNH@L-Pen (**c**,**d**).

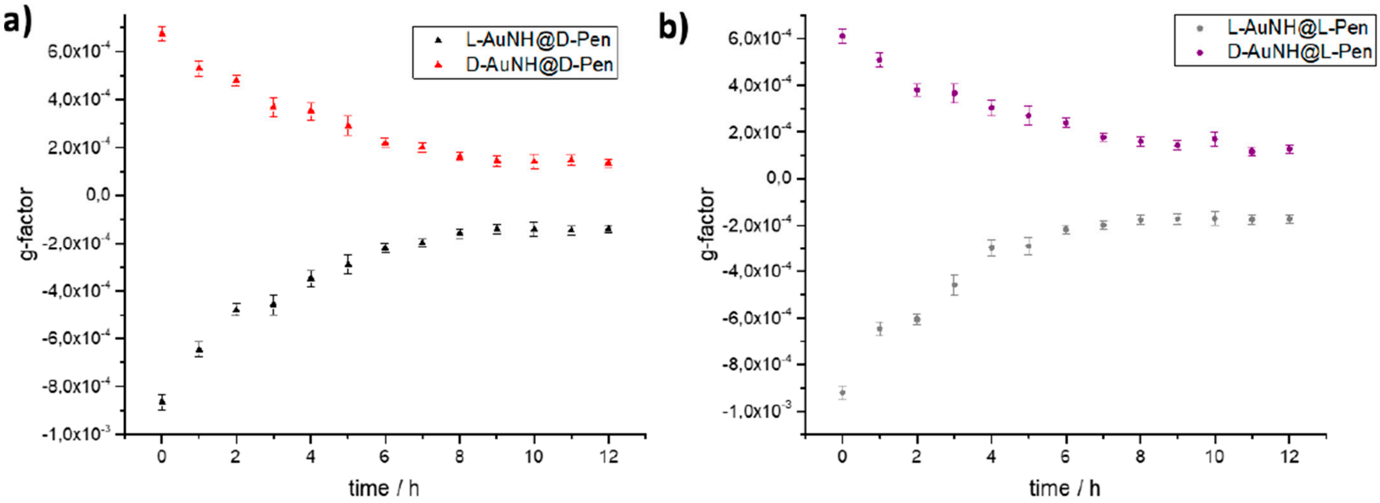

Figure S6. g-factor variation in the capping exchange experiments using (**a**) D-penicillamine and (**b**) L-penicillamine.
